# Supplementary material for: Gender Differences in the Psychopathology of Obesity: How Relevant Is the Role of Binge Eating Behaviors?
Source: Brain Sci. 2022 Jul 21;12(7):955. doi: 10.3390/brainsci12070955 (PMC9321462; doi:10.3390/brainsci12070955)
Supplement: Supplementary file 1 [file brainsci-12-00955-s001.zip › brainsci-1798109-supplementary.pdf]

Table S1. Demographic Data and Psychometric Measures.

	Total Sample (N=273)	Male Subjects (N=73)	Female Subjects (N=200)	Gender-related differences p value	No Binge Eating Disorder	Mild Binge Eating Disorder	Severe Binge Eating Disorder	Binge Eating-related differences p-value
**Demographic Data**								
**Gender**								0.059[1]
M	73 (26.73%)	-	-		49 (32.0%)	16 (22.9%)	8 (16.0%)	
F	200 (73.26%)	-	-		104 (68.0%)	54 (77.1%)	42 (84.0%)	
**Age, yrs**				0.927[2]				0.750[2]
Mean (SD)	42.08 (10.92)	42.18 (10.78)	42.04 (10.99)		42.49 (10.25)	41.31 (11.67)	41.89 (11.92)	
Range	18.62 - 71.00	19.00 - 71.00	18.62 - 66.00		18.62 - 71.00	19.00 - 68.00	19.53 - 66.00	
**Occupational Status**				<0.001[1]				0.185[1]
N-Miss	2	0	2		1	0	1	
Employed	159 (58.7%)	67 (91.8%)	92 (46.5%)		88 (57.9%)	46 (65.7%)	25 (51.0%)	
Unemployed	31 (11.4%)	5 (6.8%)	26 (13.1%)		23 (15.1%)	5 (7.1%)	3 (6.1%)	
Homeworker	74 (27.3%)	0 (0.0%)	74 (37.4%)		38 (25.0%)	17 (24.3%)	19 (38.8%)	
Student	7 (2.6%)	1 (1.4%)	6 (3.0%)		3 (2.0%)	2 (2.9%)	2 (4.1%)	
**Education, yrs of**				0.465[2]				0.116[2]
Mean (SD)	10.89 (3.50)	11.15 (3.62)	10.80 (3.46)		11.28 (3.56)	10.34 (3.22)	10.48 (3.63)	
Range	2.00 - 19.00	5.00 - 18.00	2.00 - 19.00		5.00 - 18.00	5.00 - 19.00	2.00 - 18.00	
**Body Mass Index (BMI, kg/m^2)**				<0.001[2]				0.610[2]
Mean (SD)	41.80 (7.06)	44.13 (7.36)	40.95 (6.77)		41.69 (7.37)	41.43 (7.18)	42.67 (5.87)	
Range	28.39 - 65.00	31.79 - 63.17	28.39 - 65.00		28.39 - 65.00	31.00 - 59.37	31.90 - 58.50	
**Psychometric Measures: Eating Disorders**								
**BES**				0.008[2]				< 0.001[2]
Mean (SD)	16.59 (9.45)	14.08 (8.64)	17.50 (9.59)		9.71 (4.82)	21.13 (2.58)	31.30 (4.05)	
Range	0.00 - 41.00	0.00 - 36.00	0.00 - 41.00		0.00 – 17.00	18.00 - 26.00	27.00 – 41.00	
**Binge Eating Severity**				0.0591				
Absent	153 (56.0%)	49 (67.1%)	104 (52.0%)		-	-	-	
Mild	70 (25.6%)	16 (21.9%)	54 (27.0%)		-	-	-	
Severe	50 (18.3%)	8 (11.0%)	42 (21.0%)		-	-	-	
**EDE-Q, Total Score**				<0.001[2]				< 0.001[2]
Mean (SD)	2.76 (1.07)	2.28 (1.06)	2.94 (1.01)		2.37 (1.00)	3.08 (0.87)	3.52 (0.97)	

Range	0.00 - 5.12	0.26 - 4.39	0.00 - 5.12		0.00 - 5.12	0.98 - 4.83	0.29 - 4.95	
**EDE-Q, Restrictions**				0.139[2]				0.167[2]
Mean (SD)	1.94 (1.43)	1.73 (1.32)	2.02 (1.47)		1.82 (1.45)	2.21 (1.53)	1.93 (1.18)	
Range	0.00 - 6.00	0.00 - 4.80	0.00 - 6.00		0.00 - 5.00	0.00 - 6.00	0.20 - 6.00	
**EDE-Q, Dietary**				<0.001[2]				< 0.001[2]
Mean (SD)	2.03 (1.39)	1.48 (1.20)	2.23 (1.40)		1.37 (1.07)	2.50 (1.14)	3.38 (1.32)	
Range	0.00 - 6.00	0.00 - 4.60	0.00 - 6.00		0.00 - 4.80	0.00 - 5.80	0.20 - 6.00	
**EDE-Q, Weight**				<0.001[2]				< 0.001[2]
Mean (SD)	3.25 (1.23)	2.74 (1.16)	3.43 (1.20)		2.89 (1.25)	3.45 (0.83)	4.06 (1.19)	
Range	0.00 - 6.00	0.60 - 5.60	0.00 - 6.00		0.00 - 6.00	1.20 - 6.00	0.00 - 6.00	
**EDE-Q, Shape**				<0.001[2]				< 0.001[2]
Mean (SD)	3.83 (1.44)	3.16 (1.54)	4.08 (1.33)		3.39 (1.48)	4.16 (1.12)	4.73 (1.18)	
Range	0.00 - 6.00	0.25 - 5.88	0.00 - 6.00		0.00 - 5.88	1.00 - 6.00	0.75 - 6.00	
**BUT, Global Score**				<0.001[2]				< 0.001[2]
Mean (SD)	2.09 (1.15)	1.43 (1.02)	2.33 (1.10)		1.71 (1.11)	2.40 (0.95)	2.84 (1.03)	
Range	0.00 - 4.59	0.00 - 3.82	0.00 - 4.59		0.00 - 4.41	0.00 - 4.59	0.00 - 4.56	
**BUT, Positive Symptoms**				<0.001[2]				< 0.001[2]
Mean (SD)	13.90 (8.42)	10.27 (8.57)	15.23 (7.97)		11.95 (7.64)	16.36 (8.02)	16.47 (9.74)	
Range	0.00 - 37.00	0.00 - 37.00	0.00 - 37.00		0.00 - 32.00	1.00 - 37.00	0.00 - 37.00	
**BUT, Positive Symptoms Distress**				<0.001[2]				0.006[2]
Mean (SD)	2.92 (1.03)	2.43 (1.01)	3.10 (0.98)		2.82 (1.07)	2.85 (0.96)	3.35 (0.90)	
Range	1.00 - 5.00	1.00 - 4.50	1.00 - 5.00		1.00 - 5.00	1.00 - 5.00	1.00 - 5.00	
**BUT, Weight Phobia**				<0.001[2]				< 0.001[2]
Mean (SD)	2.54 (1.33)	1.82 (1.21)	2.80 (1.28)		2.15 (1.36)	2.92 (1.07)	3.19 (1.17)	
Range	0.00 - 5.00	0.00 - 4.62	0.00 - 5.00		0.00 - 5.00	0.00 - 5.00	0.00 - 5.00	
**BUT, Body Image Concerns**				<0.001[2]				< 0.001[2]

Mean (SD)	2.86 (1.32)	2.20 (1.34)	3.11 (1.22)		2.50 (1.38)	3.17 (1.03)	3.54 (1.08)	
Range	0.00 - 5.00	0.00 - 5.00	0.00 - 5.00		0.00 - 4.89	0.00 - 5.00	0.00 - 5.00	
**BUT, Avoidance**				<0.001[2]				< 0.001[2]
Mean (SD)	1.56 (1.31)	0.92 (1.02)	1.80 (1.32)		1.12 (1.13)	1.85 (1.21)	2.50 (1.36)	
Range	0.00 - 5.00	0.00 - 4.50	0.00 - 5.00		0.00 - 4.17	0.00 - 5.00	0.00 - 4.67	
**BUT, Compulsive Self-Monitoring**				<0.001[2]				< 0.001[2]
Mean (SD)	1.21 (0.99)	0.69 (0.77)	1.40 (1.00)		0.94 (0.96)	1.39 (0.88)	1.80 (0.91)	
Range	0.00 - 5.00	0.00 - 3.40	0.00 - 5.00		0.00 - 4.00	0.00 - 5.00	0.00 - 3.60	
**BUT, Depersonalization**				<0.001[2]				< 0.001[2]
Mean (SD)	1.60 (1.28)	0.87 (1.04)	1.87 (1.26)		1.14 (1.12)	1.94 (1.19)	2.54 (1.23)	
Range	0.00 - 4.50	0.00 - 4.00	0.00 - 4.50		0.00 - 4.17	0.00 - 4.50	0.00 - 4.50	
**Psychometric Measures: Psychopathology and Quality of Life**								
**ORWELL**				0.001[2]				< 0.001[2]
Mean (SD)	54.53 (29.45)	44.95 (23.55)	58.05 (30.65)		42.33 (23.49)	64.96 (29.05)	77.73 (27.46)	
Range	1.00 - 141.00	3.00 - 110.00	1.00 - 141.00		1.00 - 121.00	12.00 - 141.00	3.00 - 135.00	
**SCL90R, Global Score Index**				0.004[2]				< 0.001[2]
Mean (SD)	0.79 (0.60)	0.61 (0.50)	0.85 (0.62)		0.57 (0.44)	0.97 (0.63)	1.22 (0.67)	
Range	0.00 - 3.13	0.04 - 2.28	0.00 - 3.13		0.00 - 2.58	0.21 - 3.13	0.14 - 2.80	
**SCL90R, Positive Symptoms**				0.004[2]				< 0.001[2]
Mean (SD)	38.74 (21.44)	32.63 (21.24)	40.97 (21.13)		30.92 (19.00)	46.31 (19.39)	52.04 (21.20)	
					0.00 - 86.00	14.00 - 88.00	8.00 - 87.00	
**SCL90R, Positive Symptom Distress**				0.958[2]				0.001[2]
Mean (SD)	2.46 (2.86)	2.47 (3.71)	2.45 (2.49)		1.89 (2.06)	3.11 (4.05)	3.26 (2.59)	
Range	0.08 - 25.62	0.08 - 25.62	0.13 - 24.00		0.08 - 15.14	0.57 - 25.62	0.33 - 11.56	
**SCL90R, Somatic Symotims**				0.002[2]				< 0.001[2]
Mean (SD)	1.08 (0.73)	0.85 (0.57)	1.16 (0.77)		0.86 (0.61)	1.35 (0.77)	1.36 (0.81)	
Range	0.00 - 3.67	0.08 - 2.42	0.00 - 3.67		0.00 - 2.83	0.00 - 3.67	0.08 - 3.33	

**SCL90R, Obsessive-Compulsive**				<0.001[2]				< 0.001[2]
Mean (SD)	0.87 (0.69)	0.64 (0.55)	0.95 (0.72)		0.63 (0.56)	1.01 (0.66)	1.41 (0.73)	
Range	0.00 - 3.10	0.00 - 2.30	0.00 - 3.10		0.00 - 2.90	0.10 - 3.10	0.00 - 3.00	
**SCL90R, Interpersonal Sensitivity**				<0.001[2]				< 0.001[2]
Mean (SD)	0.92 (0.82)	0.61 (0.67)	1.03 (0.85)		0.61 (0.60)	1.12 (0.88)	1.58 (0.88)	
Range	0.00 - 3.67	0.00 - 3.00	0.00 - 3.67		0.00 - 2.67	0.00 - 3.67	0.00 - 3.56	
**SCL90R, Depression**				0.001[2]				< 0.001[2]
Mean (SD)	0.91 (0.78)	0.66 (0.58)	1.00 (0.83)		0.65 (0.60)	1.12 (0.81)	1.42 (0.91)	
Range	0.00 - 3.69	0.00 - 2.54	0.00 - 3.69		0.00 - 2.92	0.15 - 3.69	0.08 - 3.23	
**SCL90R, Anxiety**				0.021[2]				< 0.001[2]
Mean (SD)	0.68 (0.66)	0.53 (0.51)	0.73 (0.69)		0.47 (0.50)	0.87 (0.70)	1.06 (0.76)	
Range	0.00 - 3.40	0.00 - 2.40	0.00 - 3.40		0.00 - 3.10	0.00 - 3.20	0.10 - 3.40	
**SCL90R, Hostility**				0.168[2]				< 0.001[2]
Mean (SD)	0.60 (0.65)	0.51 (0.60)	0.63 (0.67)		0.38 (0.40)	0.76 (0.67)	1.06 (0.90)	
Range	0.00 - 3.83	0.00 - 2.83	0.00 - 3.83		0.00 - 1.83	0.00 - 3.33	0.00 - 3.83	
**SCL90R, Phobia**				0.029[2]				< 0.001[2]
Mean (SD)	0.35 (0.51)	0.23 (0.42)	0.39 (0.53)		0.20 (0.35)	0.46 (0.60)	0.64 (0.61)	
Range	0.00 - 2.71	0.00 - 2.00	0.00 - 2.71		0.00 - 2.14	0.00 - 2.71	0.00 - 2.43	
**SCL90R, Paranoid Ideation**				0.005[2]				< 0.001[2]
Mean (SD)	0.80 (0.72)	0.60 (0.57)	0.87 (0.75)		0.57 (0.59)	0.93 (0.73)	1.30 (0.77)	
Range	0.00 - 3.50	0.00 - 2.33	0.00 - 3.50		0.00 - 2.83	0.00 - 3.50	0.00 - 3.00	
**SCL90R, Psychoticism**				0.249[2]				< 0.001[2]
Mean (SD)	0.46 (0.58)	0.39 (0.54)	0.48 (0.59)		0.28 (0.41)	0.58 (0.67)	0.81 (0.70)	
Range	0.00 - 3.40	0.00 - 2.40	0.00 - 3.40		0.00 - 2.30	0.00 - 3.30	0.00 - 3.40	
**SCL90R, Other**				0.931[2]				< 0.001[2]
Mean (SD)	1.01 (0.65)	1.00 (0.70)	1.01 (0.63)		0.84 (0.57)	1.17 (0.65)	1.31 (0.73)	
Range	0.00 - 3.29	0.00 - 3.29	0.00 - 3.29		0.00 - 2.71	0.14 - 2.86	0.43 - 3.29	

EDE: Eating Disorder Examination Questionnaire; BUT: Body Uneasiness Test; BES: Binge Eating Scale; SCL90R: Revised Symptom Checklist 90; O.R.WELL: Obesity Related Well-Being; SD: standard deviation.

1. Pearson's Chi-squared test
2. Linear Model ANOVA

Table S2. The role of gender in the interactions between Psychometric Measures.

	Severe BED	Moderate BED	Gender F	Interaction Moderate BED: Gender F	Interaction Severe BED: Gender F
BED – gender – SCL90 interaction					
scl_altro ~ binge * sex	0.97 (p= 0.0001)	0.52 (p=0.0036)	0.08 (p = 0.451)	-0.26 (p = 0.210)	-0.60 (p = 0.020)
scl_gsi ~ binge * sex	0.77 (p= 0.0002)	0.37 (p= 0.0162)	0.17 (p= 0.0606)	0.01 (p= 0.9512)	-0.18 (p= 0.4348)
scl_pst ~ binge * sex	27.77 (p= 0.0002)	15.33 (p= 0.0065)	6.91 (p= 0.0410)	-0.75 (p= 0.9078)	-9.24 (p= 0.2614)
scl_psdi ~ binge * sex	2.28 (p= 0.0339)	2.20 (p= 0.0067)	0.24 (p= 0.6243)	-1.31 (p= 0.1599)	-1.13 (p= 0.3397)
scl_somat ~ binge * sex	0.43 (p= 0.1013)	0.53 (p= 0.0076)	0.26 (p= 0.0303)	-0.08 (p= 0.7336)	0.03 (p= 0.9169)
scl_ocd ~ binge * sex	0.86 (p= 0.0003)	0.43 (p= 0.0169)	0.26 (p= 0.0170)	-0.08 (p= 0.6737)	-0.15 (p= 0.5767)
scl_inter ~ binge * sex	1.25 (p= 0.0000)	0.39 (p= 0.0609)	0.31 (p= 0.0123)	0.11 (p= 0.6360)	-0.38 (p= 0.2075)
scl_dep ~ binge * sex	0.83 (p= 0.0025)	0.40 (p= 0.0501)	0.25 (p= 0.0446)	0.04 (p= 0.8486)	-0.13 (p= 0.6680)
scl_ansia ~ binge * sex	0.70 (p= 0.0027)	0.30 (p= 0.0843)	0.12 (p= 0.2482)	0.11 (p= 0.5608)	-0.16 (p= 0.5359)
scl_ost ~ binge * sex	0.62 (p= 0.0070)	0.37 (p= 0.0334)	0.02 (p= 0.8086)	0.01 (p= 0.9558)	0.06 (p= 0.7965)
scl_fob ~ binge * sex	0.56 (p= 0.0022)	0.18 (p= 0.1953)	0.09 (p= 0.2452)	0.09 (p= 0.5677)	-0.16 (p= 0.4066)
scl_par ~ binge * sex	0.73 (p= 0.0038)	0.28 (p= 0.1358)	0.17 (p= 0.1305)	0.07 (p= 0.7304)	-0.03 (p= 0.8968)
scl_psic ~ binge * sex	0.78 (p= 0.0002)	0.24 (p= 0.1206)	0.05 (p= 0.5900)	0.06 (p= 0.7232)	-0.32(p= 0.1613)
scl_altro ~ binge * sex	0.97 (p= 0.00019	0.52 (p= 0.0036)	0.08 (p= 0.4514)	-0.25 (p= 0.2101)	-0.60 (p= 0.0212)
BED – gender – EDE interaction					
ede_alim ~ binge * sex	1.75 (p=0.0001)	1.10 (p=0.0007)	0.47 (p=0.0145)	-0.03 (p=0.9339)	0.21 (p=0.6551)
ede_forma ~ binge * sex	1.90 (p=0.0002)	0.94 (p=0.0121)	0.95 (p=0.0000)	-0.34 (p=0.4264)	-0.85 (p=0.1223)
ede_peso ~ binge * sex	1.71 (p=0.0001)	0.76 (p= 0.0183)	0.74 (p=0.0002)	-0.34 (p=0.3528)	-0.79 (p= 0.0944)
ede_rest ~ binge * sex	-0.07 (p=0.8929)	0.30 (p=0.4646)	0.22 (p=0.3738)	0.08 (p=0.8513)	0.17 (p=0.7732)
ede_tot ~ binge * sex	1.32 (p=0.0003)	0.78 (p=0.0041)	0.60 (p= 0.0003)	-0.15 (p=0.6108)	-0.31 (p=0.4261)
BED – gender – BUT interaction					
but_a ~ binge * sex	1.62 (p= 0.0003)	0.62 (p= 0.0618)	0.76 (p= 0.0002)	0.04 (p= 0.9047)	-0.43 (p= 0.3737)
but_bic ~ binge * sex	1.85 (p= 0.0001)	1.02 (p= 0.0032)	1.08 (p= 0.0000)	-0.59 (p= 0.1391)	-1.18 (p= 0.0197)
but_csm ~ binge * sex	0.90 (p= 0.0084)	0.57 (p= 0.0277)	0.69 (p= 0.0000)	-0.23 (p= 0.4257)	-0.18 (p= 0.6362)
but_d ~ binge * sex	1.38 (p= 0.0011)	0.84 (p= 0.0084)	0.90 (p= 0.0000)	-0.17 (p= 0.6346)	-0.16 (p= 0.7312)
but_gsi ~ binge * sex	1.56 (p= 0.0001)	0.84 (p= 0.0035)	0.93 (p= 0.0000)	-0.31 (p= 0.3446)	-0.69 (p= 0.1032)
but_psdi ~ binge * sex	1.14 (p= 0.0024)	0.08 (p= 0.7608)	0.77 (p= 0.0000)	-0.14 (p= 0.6628)	-0.86 (p= 0.0396)
but_pst ~ binge * sex	7.46 (p= 0.0142)	5.89 (p= 0.0103)	5.56 (p= 0.0001)	-2.59 (p=0.3271)	-4.55 (p= 0.1761)
but_wp ~ binge * sex	1.72 (p= 0.0002)	0.99 (p= 0.0042)	1.08 (p= 0.0000)	-0.42 (p= 0.2857)	-1.02 (p= 0.0450)

SCL: Symptom Checklist; BED: Binge Eating Disorder; BES: Binge Eating Scale; BUT: Body Uneasiness Test; But A: BUT Avoidance Subscale; BUT D: BUT Depersonalization Subscale; GSI: Global Severity Index; PST: Positive Symptom Total; PSDI: Positive Symptom Distress Index; BIC: Body Image Concern; CSM: Compulsive Self - monitoring; WP: Weight Phobia.
